# Assessment of Climate Change Vulnerability at the Local Level: A Case Study on the Dniester River Basin (Moldova)

**DOI:** 10.1155/2013/173794

**Published:** 2013-05-20

**Authors:** Roman Corobov, Igor Sîrodoev, Sonja Koeppel, Nickolai Denisov, Ghennadi Sîrodoev

**Affiliations:** ^1^Eco-TIRAS International Environmental Association, 11A Teatrala Street, 2012 Chisinau, Moldova; ^2^University of Bucharest, Interdisciplinary Center of Advanced Researches on Territorial Dynamics, 4-12 Regina Elisabeta Boulevard, 030018 Bucharest, Romania; ^3^Institute of Ecology and Geography, Moldavian Academy of Sciences, 1 Academiei Street, 2028 Chisinau, Moldova; ^4^United Nations Economic Commission for Europe, Palace des Nations, 8-14 Avenue de Paix, 1211 Geneva 10, Switzerland; ^5^ZOI Environment Network, International Environment House II, 9, Chemin de Balexert, Châtelaine, 1219 Geneva, Switzerland

## Abstract

Vulnerability to climate change of the Moldavian part of the Dniester river was assessed as the function of exposure, sensitivity, and adaptive capacity of its basin's natural and socioeconomic systems. As a spatial “scale” of the assessment, Moldova's administrative-territorial units (ATUs) were selected. The exposure assessment was based on the climatic analysis of baseline (1971–2000) temperature and precipitation and projections of their changes in 2021–2050, separately for cold and warm periods. The sensitivity assessment included physiographical and socioeconomic characteristics, described by a set of specific indicators. The adaptive capacity was expressed by general economic and agricultural indicators, taking into consideration the medical provision and housing conditions. Through a ranking approach, the relative vulnerability of each ATU was calculated by summing its sensitivity and adaptive capacity ranks; the latter were obtained as combinations of their primary indicator ranks, arranged in an increasing and decreasing order, respectively. Due to lack of sound knowledge on these components' importance in overall assessment of vulnerability, their weights were taken as conventionally equal. Mapping of vulnerability revealed that ATUs neighboring to municipalities are the most vulnerable and need special attention in climate change adaptation. The basin's “hotspots” were discussed with public participation.

## 1. Introduction

The measurement of vulnerability to climate change is a central moment in adaptation activity to mitigate adverse climatic impacts. Both natural and social scientists try to measure and assess such vulnerability, whether from the perspective of regions, socioecological systems, or individuals. Different approaches to this issue have penetrated into climate change research, and with rapid growth of attention to vulnerability, the concept itself has been redefined, and new interpretations and approaches were developed [1–7].

Climate change represents a classic global problem characterized by infinitely diverse actors, multiple stressors, and multiple scales. As a result, research on vulnerability to this phenomenon must address at least three important challenges: (1) to improve approaches for comparing and aggregating impacts across diverse sectors and populations, (2) to model socioeconomic transformation in assessing the significance of these impacts, and (3) to account for multiple dimensions. Among the latter Moss et al. [8] named the physical-environmental impacts of a changing climate, a capacity to recover from extreme events and adapt to climate change over the longer term, and the degree to which international links and other connections assist a region (country) in its coping and adaptive efforts. At the same time, the existence of competing conceptualizations and terminologies of vulnerability is particularly problematic since they are characterized by intense collaboration between scholars from different scientific schools of thought, including the physical science, risk assessment, sustainable development, economics, and policy analysis. Different definitions not only result in different “diagnoses” of the problem but also in different kinds of “cures” [6].

Initially, the assessment of vulnerability to climate change was approached from an impact's point of view where vulnerability was defined as “…the degree to which a system is susceptible to, and unable to cope with, adverse effects of climate change, including climate variability and extremes. Vulnerability is a function of the character, magnitude, and rate of climate change and variation to which a system is exposed, its sensitivity, and its adaptive capacity” [9, page 883]. This approach continues to base many of today's assessments and adaptation prioritization efforts. However, the emphasis in these efforts has recently moved from better defining exposure and potential impacts to a better understanding of factors, which affect sensitivity of societies to these impacts and their capacity to adapt. There is an increasing recognition of the importance to consider the social vulnerability equally with the biophysical vulnerability, thus presenting vulnerability on the whole as a function both of physical characteristics of climate change and of social systems' inherent sensitivity and adaptive capacity. Various researchers have tried to bridge the gap between the social, natural, and physical sciences' contributions to new methodologies that confront this challenge. The attempts to bridge all approaches are especially advanced under the umbrella of sustainability and resilience [4, 10–14]. 

Also, the vulnerability analysis, being a useful integrative and multidimensional conception for evaluating the potential effects of climate change, is a very complex issue unlikely to be directly observed and measured. First of all, due to interactions between socioenvironmental systems and climate conditions, one cannot describe exhaustively the impacts of climate change. Yet there is also no consensus as to what indicators to measure these impacts and, even within a given conceptual framework for considering vulnerability, other questions arise in the practice of vulnerability assessment. 

A system of vulnerability “measurement” is usually developed to allow comparisons between different places, social groups, or sectors whose vulnerabilities are not static but respond to changes in physical, economic, social, political, or institutional conditions over time [15]. Different vulnerability indices and indicators were designed to better understand the drivers of vulnerability or to provide the abovementioned comparison in terms of the climate change risks and capacities to deal with them. The main challenges in selecting representative criteria of vulnerability at any level derive from the fact that effects of climate-induced pressures are mediated by society. Consequences vary between communities, social groups in a community, individual households, and even between people within a household. A common thread is an attempt to quantify multidimensional issues, using indicators as proxies. These are often combined into a composite index allowing diverse variables to be integrated (e.g., [16]). Nevertheless, even such integrated indexes are developed to address some specific tasks, and they cannot be considered as a universal measure of vulnerability. In this regard, the task, assigned to the present study, has required some modifications of the general conceptual framework of vulnerability assessment.

The *main goal* of the research was to assess vulnerability of Dniester river basin to climate change on the local level through addressing two specific tasks: (1) to develop and practically realize a methodology for assessing the climate change vulnerability at the level of second-tier administrative-territorial units (ATUs) of Moldova and (2) to build spatial models of the vulnerability of the Moldavian part of the Dniester river basin. 

## 2. Materials and Methods

Usually, the assessment of vulnerability to any phenomenon requires a clear conceptual framework. The most widely used approach to the assessment of climate change vulnerability is based on the above mentioned definition, proposed by the IPCC [9], and focuses on its three components: exposure to climate stressors, climate sensitivity, and adaptive capacity.

In this triad, the exposure refers to the degree of climate impacts on certain system that can be represented by long-term observations of a regional local climate and its expected change, including climate variability and extremes. Sensitivity defines the degree, to which the system is susceptible to direct or indirect climatic impacts. Finally, adaptive capacity describes the capability of a system to adapt to real or expected climatic stresses and to cope with their consequences. Exposure is usually treated as an external dimension of vulnerability, while sensitivity and adaptive capacity represent its internal dimension. A highly vulnerable system would be very sensitive to modest changes in climate, while its capabilities to cope with significant negative effects are limited [17].

There is no unique methodology of vulnerability assessment; it has to fit for specific objects and evaluation tasks. However, in the context of water-related issues of a river basin such an assessment undoubtedly should serve the purposes of its sustainable management. At the same time, vulnerability is influenced not just by available water resources, both present and future, but also by the whole range of social, economic, and environmental factors, which determine the ability of the basin to cope with changing external conditions.

To take into consideration a temporal dimension means that an assessment should distinguish between present-day and future vulnerability of a river (in our case the Dniester river) water resources. Present-day vulnerability applies to the current variability of regional climate and present ability of natural and social systems to cope with the current variability of the river's hydrological regime. Assessment of present-day vulnerability contributes to substantial understanding of potential responses to expected impacts; obviously, measures designed to increase today's adaptation potential will reduce future vulnerability. Future vulnerability relates to expected climatic conditions and future capacity to confront their adverse impacts, primarily its likely more severe and frequent extremes [18].

Provided that vulnerability of water resources depends on the combination of multiple factors, its assessment should include a series of aspects and criteria: physiographical, social, economic, environmental, adaptive, and so forth. Evidently also, the completeness of the evaluation depends on the availability and accessibility of corresponding background information. These and some other considerations have served as a basis for the methodological approaches.

**Figure 1:**
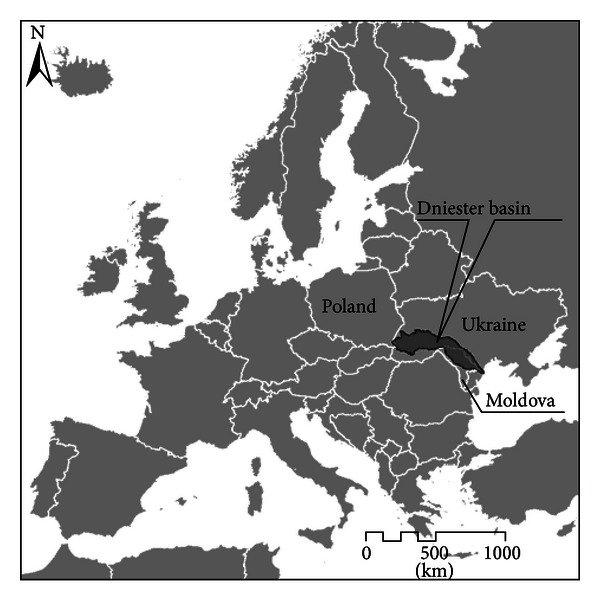
Location of the Dniester river basin in Europe.

The study area covers the Moldavian right bank part of the Dniester river (hereafter—Dniester) basin. This transboundary (between Moldova and Ukraine) river has a length of 1,352 km, of which 660 km runs in Moldova where its basin occupies about 67% of its territory (Figure 1). Being the main river of the country, Dniester plays an important role in Moldova's economy and population well-being.

As a local scale of the vulnerability assessment, 22 second-tier ATUs of Moldova were selected, including two municipalities (Chisinau and Balti) and 20 *raions* (basic administrative units of Moldova), the greater part of which is located within the Dniester basin.

**Figure 2:**
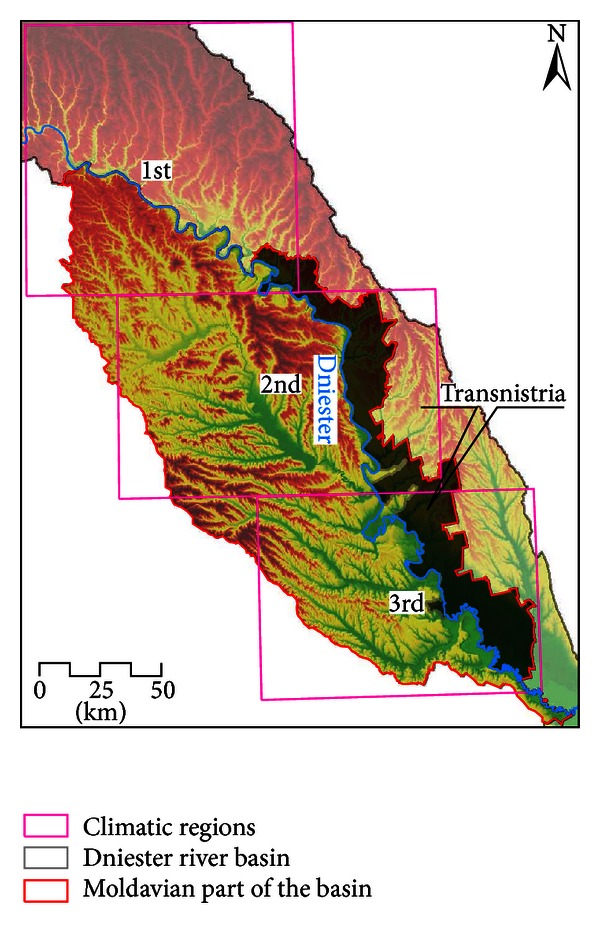
Climatic subregions for averaging the baseline and climate models' grid information used for assessment of the basin's exposure to current and future climate.

A main limitation of the assessment was objective impossibility to consider future social-economic conditions and climate change projections for each ATU: the former are in principle unpredictable in Moldova's transition economy; the latter were derived only at a coarser scale as average values for individual parts of the basin (see Figure 2). Therefore, for the climate sensitivity and adaptive capacity evaluation, the data of recent national statistical surveys [19] were used, with the assumption that most parameters would rather worsen than improve under the changed climate. Another limitation consisted in a difficulty to quantify the “weights” assigned to each factor or indicator in the integrated vulnerability assessment; this difficulty was caused by lack of a science-based monetary, expert, or other objective evaluation criteria for reliable comparison of chosen indicators.

Finally, in the case of evaluating the complex objects, such as the Dniester basin, inevitably there is a need to operate with both partial and general estimations. The importance of partial assessments results from the impossibility to evaluate a natural complex as a whole without the preliminary evaluation of its individual properties. The necessity of general assessments arises due to a fact that “utility” of an object is determined by totality of its particular properties, which is best expressed through one general index, frequently measured in rating scores or ranks. Because the general assessment of a complex system disregards individual properties of its parts, while particular assessments give no idea about the system as a whole, combining the two approaches was used in this research.

In the ranking approach, a relative vulnerability to climate change of each ATU was assessed depending on its “place” in the ranked sequence of individual components of vulnerability. In turn, the rank of each component was obtained through the combination of the ranks of primary indicators sorted in decreasing or increasing order. For some intermediate calculations, where it was possible to use expert evaluations, for example, in the case of physiographical sensitivity, certain proxies were weighted by averaging the ranks of primary indicators. The list of indicators and the evaluation scheme, consisted of sequential steps, are presented in Table 1.

## 3. Results and Discussion

### 3.1. Evaluation of Vulnerability

#### 3.1.1. Exposure Assessment

The exposure component of vulnerability evaluated new characteristics of regional climate, described as likely changes in key baseline climatic variables (air temperature and precipitation) and one applied climatic characteristic (the index of air humidity in a warm season). The assessment was based on the analysis of historical observations of temperature and precipitation in the 30-year baseline period (1971–2000) and the projections of their changes for the middle of the 21st century (2021–2050), kindly provided by Krakovskaya [20]. To develop climate change scenarios for the Dniester basin, she used outputs of seven regional climatic models (RCMs) with 25 km spatial resolution, which had been realized within the EC research project ENSEMBLES for A1B SRES scenario. Initial and boundary conditions for RCMs were taken from four global circulation models [21]. The modeled grid information for Moldavian part of the basin was averaged for three climatic subregions (Figure 2).

Because climatic threats are different for each season, there are no reasons to consider an exposure to their stressors in annual climatic variables, and its assessment was done separately for cold (November–March) and warm (April–October) seasons (Tables 2 and 3). 

The warm season assessment was mainly focused on humidity conditions as an indicator better describing the combined impact of ambient temperature and precipitation. Moreover, the significant part of the basin is located in a semiarid zone where rainfalls are the main limiting factor for crops production, and any further aridization on its territory could substantially influence the volume of available water resources and their distribution by seasons and different parts of the basin [22]. Air Humidity Index (HI), which was selected to describe humidity conditions, represents a ratio of precipitation to evapotranspiration and was calculated as a function of monthly air temperature and precipitation, using the statistical approach proposed in [23]. 

Some basic characteristics of the present-day warm season climate in the middle and lower Dniester can be outlined as follows (Table 2):

- a clear temperature increase from 14.5°C in the middle part of the river to 16.4°C at its mouth;
- a southward decrease of precipitation: from 415 mm in the middle part to 340 mm at the mouth;
- a corresponding increase in the warm season aridity caused by opposite spatial changes in temperature and precipitation reflected in the decrease of HI from 0.75 in the north part of the studied territory to 0.53 in the south.

With respect to climate change, the picture is somewhat different. The increase of average temperature by 1.0–1.5°C, bigger in the south, is obvious. However, precipitation change in this period (increase by 8 mm in the north and decrease by 6 mm in the south) can be neglected. Since temperature increase is not compensated by corresponding increase in precipitation, a growing aridity is expected, expressed in decreasing HI by 1–8%. Thus, one can *a priori *presuppose that in a warm season the southern part of the Dniester basin that already suffers from droughts and limited water resources [24] will be the most vulnerable to climate change. 

For the cold season, an air temperature regime was chosen as a main vulnerability criterion. High or low ambient temperatures, as well as their drops, influence multiple socioeconomic factors (heating and transportation costs, population morbidity and mortality, etc.). Also, a temperature regime determines conditions of crops overwinter surviving. In particular, hard frosts alternating with thaws inflict heavy damages on cereals due to winter killing and damping off. At the same time, based on the projections of likely changes (Table 3), the cold season precipitation sums will remain virtually unchanged.

On the whole, the observed spatial variability of temperature and humidity in the middle and lower Dniester indicated certain differences in exposure to climatic conditions of individual ATUs, thus giving some justification for their ranking by the exposure. However, for a number of reasons, this procedure could not be realized. In particular,

- available information allows evaluating the climatic conditions of the study area by three climatic regions, and therefore, each of the 22 administrative units can be only attributed to one of the ranks;
- effects of temperature conditions of the two seasons are opposite. In the warm season, southward temperature increase leads to increasing aridity, thus intensifying the negative exposure to climatic conditions. In the cold period, on the contrary, higher temperatures in the south create more favorable conditions, thus somewhat “compensating” here a greater vulnerability to climatic conditions of the warm period;
- lack of science-based criteria for evaluating the effects of each of these two factors makes it extremely difficult to assign them appropriate weights; this limitation of the assessment results in a zero net effect when two seasons are combined. To remove this artifact the separate evaluation of temperature and humidity regimes for each administrative unit is needed. Such an approach could provide a more differentiated ranking of exposures and eliminate mutual exclusion of the ranks.

As a result, the exposure was excluded as a component in the overall climate change vulnerability assessment of the Dniester basin at a local level, limiting the assessment to comparison of the basin's sensitivity and adaptive capacity.

### 3.2. Sensitivity Assessment

As it was shown in Table 1, the sensitivity assessment included two blocks: physiographical and social-economic; in turn, each of these blocks was described by a set of indicators (Table 4).

In the *physiographical block* a land use structure, expressed as percentages of its individual indicators in an ATU's area, defines environmental sensitivity, mainly an anthropogenic load on the land. It is supposed that territories, where arable lands dominate, are more subjected to climate risks and thus are more sensitive than, for example, lands under perennial plants, grasslands, pastures, and forests. Also, the larger the built-up area (or the level of urbanization), the higher the physical sensitivity and, respectively, vulnerability of the territory. Soil degradation and geomorphologic processes (ravines, surface erosion, and landslides) determine soil quality and ecological conditions. In these assessments, all indicators in a block (subblock) are treated as independent, and the ranking by a particular indicator implied equality of the rest. According to such an approach, Făleşti is the most sensitive *raion* from the physiographical point of view, while Ocniţa is the least sensitive one.

In the *socioeconomic block,* four aspects were evaluated, each of which also formed a kind of subblock. The resulting sensitivity can be described as follows.

- In the demographic subblock, a population density is considered as a representative indicator of populations' general sensitivity to climatic threats. It is supposed that relatively more populated ATUs are more vulnerable as compared to the less populated. Sensitivity also increases in parallel with the increasing share of urban and female populations, which are, according to numerous publications, for example, [9], among the most vulnerable categories. Growth of a demographic load, described as a ratio of incapacitated population to the able-bodied part of a society, indirectly increases its vulnerability. The negative natural population growth in present-day Moldova, when death rate exceeds birth rate, also increases sensitivity.
- The agricultural subblock was chosen as a main evaluation criterion of economic sensitivity due to the role of agriculture in Moldavian economy and rural employment. Undoubtedly, regions with, for example, low crop yields or reduced productivity in cattle-farming (e.g., yield of milk), are more sensitive than those where these indicators are higher.
- High unemployment and crime rates also contribute to increasing sensitivity to climate change effects.

By their physiographical condition, Făleşti *raion* is the most sensitive to external stresses, and Ocniţa *raion* is the least sensitive. According to the level of socioeconomic development, Dubăsari *raion* is the most sensitive ATU, while Sîngerei *raion* is at an opposite side of the hierarchy. Densely populated and highly urbanized municipalities Chişinău and Bălţi are also highly sensitive in socioeconomic terms. According to the combined physiographical and social-economic factors, Soroca *raion* seems to be the most sensitive, and Teleneşti *raion* is the least sensitive to climate change.

### 3.3. Assessment of the Adaptive Capacity

Adaptive capacity was evaluated as the function of a set of general economic and agricultural indicators, as well as taking into consideration the state of medical provision and housing conditions (see Table 1). Obviously, the higher the levels of each of these indicators in a certain ATU, the higher its adaptive capacity to climate change; the sum of indicators' ranks determines its adaptive capacity relative to other ATUs. As it would be expected, the capital city Chişinău has the highest adaptive potential to confront climate change challenges, while Dubăsari *raion's* potential is the smallest (Table 5).

### 3.4. Vulnerability Assessment

Vulnerability to climate change was calculated by summing up the sensitivity and adaptive capacity ranks. Due to the aforementioned lack of a clear knowledge on importance of these two components in the overall assessment of vulnerability, their weights were taken as conventionally equal. In the process of adding two sides of vulnerability, the sensitivity was ranked in a decreasing order, and the adaptive capacity was sorted increasingly. The resulted ATUs' ranks of overall vulnerability are shown in Table 6.

As it is seen from this table, Rezina *raion* has the highest relative vulnerability in comparison with other regions of the basin, being among the first ATUs both with high sensitivity and with low adaptive capacity. Floreşti is the *raion* with the lowest vulnerability; its relatively low sensitivity is combined with a high adaptive capacity.

## 4. Vulnerability Mapping

The maps of vulnerability as well as of its components provide information that can lead to climate change impacts reduction through safety and environmentally conscious policies and practices. Numerous vulnerability maps are being prepared (e.g., [25–27]) to better understand the drivers of vulnerability and to compare countries, regions, communities, and so forth in terms of the risks they face from climate change consequences and their capacity to deal with them. The comparison of vulnerabilities facilitates allocation of limited resources equitably and efficiently among different entities—regions, administrative groups, or different proponents of adaptation; this is especially important for transition economies, to which Moldova is attributed.

**Figure 3:**
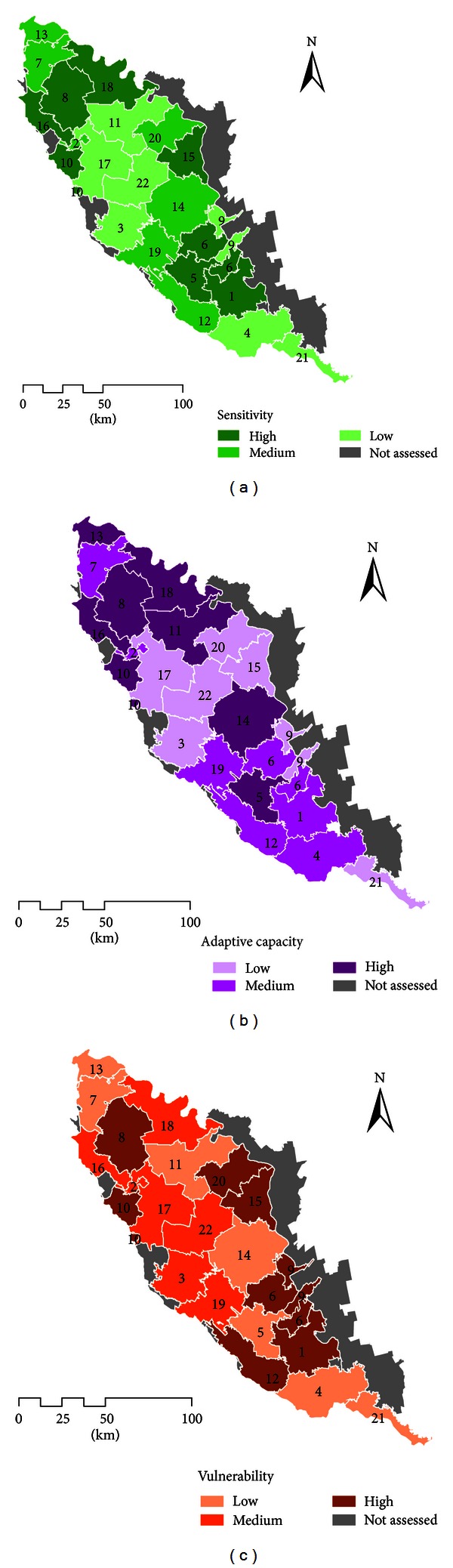
Vulnerability to climate change (c) of Moldova's administrative-territorial units as a function of their sensitivity (a) and adaptive capacity (b). Notes: (1) numbering of ATUs is made according to Table 4; (2) as “not assessed” there are shown raions of Transnistria (due to the lack of an access to relative information in this politically separative region) and those Moldavian west raions, which have just small parts of their territory within the basin.

The spatial assessment of sensitivity, adaptive capacity, and vulnerability of the Moldavian part of Dniester basin is shown in Figure 3. To address this task, all ranked series (Tables 4–6) were divided into three categories collating the administrative-territorial units with high, medium, and low values of evaluated parameters. Thus, resulted maps highlight areas, which are needed in the first priority adaptation measures. In particular, almost all *raions* neighboring to Chişinău municipality are among the most vulnerable and require special attention in policies dealing with climate change adaptation.

## 5. Participatory Analysis of the Assessment's Results

**Figure 4:**
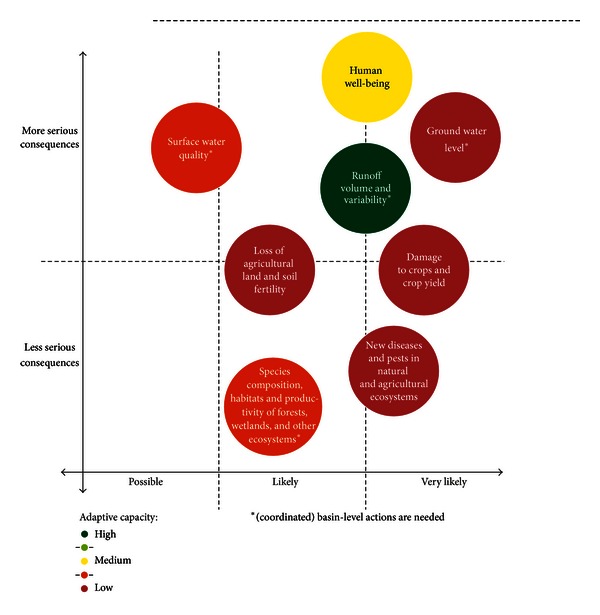
Graphical summary of the participatory analysis of inputs to the Dniester basin vulnerability to climate change according to the severity of consequences and their probability (design by Carolyne Daniel, Zoï environment network, Geneva).

To make the study's findings well known and to test them in the real-life context, there were organized wide consultations with people, communities, and organizations of the Dniester basin. Preliminarily, based on the research outputs the short textual (Table 7) and graphical (Figure 4) summaries describing the main identified risks, their probability and urgency, as well as current adaptive capacity were developed and presented at a specially organized workshop. During a facilitated discussion, the representatives of ATUs, sectoral ministries and agencies, academic, and nongovernmental organizations reviewed the findings from their regional or sectoral perspectives, commented the conclusions, and suggested priority adaptation measures to reduce likely climate-related risks. It was also discussed which of the proposed measures can be taken on the country level or locally, and which of them require cross-border cooperation or coordination with the neighbors. 

On the whole, this exercise has achieved several goals:

- promoted the communication of the vulnerability assessment findings to a broad and diverse audience in the basin;
- “confirmed” them (or not, as the case may be) from a largely nonacademic perspective;
- provided a platform for brainstorming about specific options for adaptation.

A methodological conclusion from the carried out consultations is that short and simple summaries of the vulnerability assessment are very effective tools for a real public participation in dealing with such a complex issue as the assessment of climate change vulnerability at a river basin's level. People were happy not only to move “bubbles” on the graph or to change their colors from the viewpoint of the impacts intensity and probability but eventually were able to discuss the issues in-depth and much better understand their content, context, and interdependence.

## 6. Conclusion

The approach, used in this work for the so-called “quick” assessment of present-day vulnerability to climate change that was based on the historical and up-to-date statistical data, has demonstrated undoubted efficiency, when applied at the local level. Nevertheless, the term “quick,” borrowed from Yusuf and Francisco [27], underlines an idea that such assessments should be considered just as the first experience, highly dependent on available information and heavily relying on expert evaluation. 

Also, it is obvious that the carried out vulnerability assessment has to be extended and deepened along with progress in climate change scenarios development and increasing knowledge in the studies of complex interactions of the environment and socioeconomic development. At the same time, it is necessary to further identify and collect detailed information on various indicators of sensitivity and adaptive capacity at the local level. It requires more time and resources, but only in this case you can reduce uncertainties that continue to prevail in this issue. Such improvements will be the subject of new national and international researches aimed at more efficient use of information as well as of methods to select and interpret the most representative vulnerability indicators. 

A participatory analysis in the vulnerability assessment, with involving the broad range of stakeholders from the river basin, has proved to be an effective tool for both verifying and better communicating the assessment results.

## Figures and Tables

**Table 1:** Evaluation scheme of the assessment of vulnerability to climate change.

Sector	Indicator		Functional relationships	Individual and average weights			
Exposure							

	Temperature change in a warm season		Temperature↑ exposure↑	0.25		**0.5**
Climate	Humidity index in a warm season		Humidity index↓ exposure↑	0.25		
	Temperature change in a cold season		Temperature↑ exposure↓			**0.5**	

Sensitivity							

*Physiographical sensitivity *							

	Arable land		Area↑ sensitivity↑	2.0			
	Perennial plants			1.0			
Land use (%)	Grasslands		Area↑ sensitivity↓	1.5	**0.25**	***0.33 ***	
	Forests			2.0			
	Surface water			2.0			
	Soil quality		Quality↓ sensitivity↑		**0.25**		**−0.5**
		Surface erosion			**0.25**		
Soils	Geomorphologic processes	Ravines	Area↑ sensitivity↑	1.0	**0.25**	***0.33 ***	
		Landslides		2.0			
Construction	Built-up areas					***0.33 ***	

*Social-economic sensitivity *							

	Population density (no. of inhabitants per sq. km)		Density↑ sensitivity↑		**0.20**		
	Urban population (%)		Share↑ sensitivity↑		**0.20**		
Population	Women (%)				**0.20**	**0.25**	
	Natural growth		Growth↓ sensitivity↑		**0.20**		
	Demographic load		Load↑ sensitivity↑		**0.20**		
	Ratio of unprofitable versus profitable enterprises		Ratio↑ sensitivity↑		**0.17**		
	Annual average yield of milk				**0.17**		**0.5**
Agriculture		potatoes			**0.17**		
	Yields	vegetables	Yield↓ sensitivity↑		**0.17**	**0.25**	
		fruits			**0.17**		
		cereals			**0.17**		
Labor force	Unemployment rate					**0.25**	
Crime rate	Total crime rate		Rate↑ sensitivity↑	0.5		**0.25**	
	Grave crimes			0.5		

Adaptive capacity							

	Road density		Density↑ capacity↑		**0.20**		
	Share of industrial workers		Share↑ capacity↑		**0.20**		
Economics	Mobility of employees		Mobility↑ capacity↑		**0.20**	**0.25**	
	Investments in capital asset		Investments↑ capacity↑		**0.20**		
	Average monthly wage		Wage↑ capacity↑		**0.20**		
	Milk production		Production↑ capacity↑		**0.33**		
Agriculture	Slaughter of cattle and poultry				**0.33**	**0.25**	**0.5**
	Use of mineral fertilizers (per 1 ha)		Optimal use↑ capacity↑		**0.33**		
	No. of physicians per 10 thou. inhabitants				**0.33**		
Medical provision	No. of middle medical staff per 10 thou. inhabitants		Number↑ capacity↑		**0.33**	**0.25**	
	No. of beds in hospitals per 10 thou. inhabitants				**0.33**		
Housing	Building of new houses		Housing↑ capacity↑		**0.5**	**0.25**	
	Housing provision rate				**0.5**	

↑, ↓: increase or decrease of an indicator.

**Table 2:** Temperature and precipitation conditions of a warm season in the Moldavian part of the Dniester basin.

Month	Climatic subregions								
	1st			2nd			3rd		
	*T* (°C)	*P* (mm)	HI	*T* (°C)	*P* (mm)	HI	*T* (°C)	*P* (mm)	HI
Baseline period (1971–2000)									

April	8.8	48	0.79	9.8	43	0.65	10.0	39	0.57
May	14.7	59	0.70	15.6	51	0.56	16.0	49	0.51
June	17.9	84	0.83	19.1	75	0.64	19.7	61	0.43
July	19.2	85	0.87	20.5	73	0.67	21.4	58	0.45
August	18.7	55	0.84	20.0	54	0.48	21.0	49	0.38
September	14.1	55	0.85	15.3	55	0.79	16.3	50	0.66
October	8.4	29	0.69	9.5	32	0.71	10.4	34	0.72
**Season**	**14.5**	**415**	**0.75**	**15.7**	**383**	**0.64**	**16.4**	**340**	**0.53**

Projected period (2021–2050)									

April	9.7	53	0.82	10.9	48	0.66	11.1	41	0.54
May	15.4	63	0.72	16.8	53	0.53	17.2	50	0.47
June	18.8	88	0.82	20.7	76	0.55	21.4	61	0.32
July	19.8	82	0.81	22.1	70	0.55	23.0	54	0.32
August	19.6	50	0.45	21.8	48	0.34	22.9	41	0.22
September	15.1	61	0.90	16.9	59	0.78	18.0	55	0.66
October	9.9	26	0.54	11.2	29	0.55	12.3	32	0.58
**Season**	**15.5**	**423**	**0.72**	**17.2**	**383**	**0.57**	**18.0**	**334**	**0.43**

**Table 3:** Temperature and precipitation conditions of a cold season in the Moldavian part of the Dniester basin.

	Climatic subregions					
Month	1st		2nd		3rd	
	*T* (°C)	*P* (mm)	*T* (°C)	*P* (mm)	*T* (°C)	*P* (mm)
Baseline period (1971–2000)						

November	2.2	37	3.2	37	4.2	38
December	−1.5	35	−0.7	34	0.1	36
January	−3.8	27	−2.9	28	−2.1	29
February	−2.6	27	−1.7	27	−0.9	29
March	1.8	29	2.8	28	3.2	28
**Season**	**−0.8**	**155**	**0.1**	**154**	**0.9**	**160**

Projected period (2021–2050)						

November	3.2	36	4.3	36	5.3	39
December	−0.2	37	0.8	35	1.6	35
January	−2.4	29	−1.5	29	−0.6	30
February	−0.9	25	0.2	25	0.9	27
March	2.8	32	4.1	30	4.6	29
**Season**	**0.5**	**159**	**1.6**	**155**	**2.36**	**160**

**Table 4:** Ranks of Moldova's administrative-territorial units in the decreasing order of their sensitivity.

No.	ATU	Sensitivity									
		Physiographical				Social-economic					Total rank
		Indicator's rank^a^			Intermediate rank	Indicator's rank^b^				Intermediate rank
		a1	a2	a3		b1	b2	b3	b4	
(1)	*Anenii Noi *	15	9	11	***10 ***	3	11	22	9	***10 ***	**7**
(2)	*Bălţi *	14	21	1	***12 ***	2	5	19	1	***2 ***	**10**
(3)	*Călăraşi *	20	1	13	***9 ***	6	15	14	21	***18 ***	**16**
(4)	*Căuşeni *	8	12	20	***16 ***	22	8	7	3	***9 ***	**18**
(5)	*Chişinău *	18	18	2	***15 ***	4	4	18	2	***3 ***	**5**
(6)	*Criuleni *	4	17	6	***6 ***	8	3	15	12	***6 ***	**6**
(7)	*Donduşeni *	3	19	19	***17 ***	14	21	4	17	***17 ***	**15**
(8)	*Drochia *	1	14	10	***4 ***	9	13	12	13	***11 ***	**3**
(9)	*Dubăsari *	9	20	9	***14 ***	1	6	1	15	***1 ***	**20**
(10)	*Făleşti *	2	11	3	***1 ***	16	14	5	20	***16 ***	**2**
(11)	*Floreşti *	17	8	18	***20 ***	12	18	10	20	***19 ***	**17**
(12)	*Ialoveni *	22	2	7	***8 ***	10	7	13	8	***8 ***	**9**
(13)	*Ocniţa *	11	22	17	***22 ***	7	22	11	10	***13 ***	**11**
(14)	*Orhei *	21	6	16	***18 ***	5	17	21	19	***20 ***	**14**
(15)	*Rezina *	12	4	8	***2 ***	19	10	9	14	***15 ***	**8**
(16)	*Rîşcani *	6	10	12	***7 ***	13	16	6	16	***14 ***	**4**
(17)	*Sîngerei *	19	3	14	***13 ***	20	19	16	11	***22 ***	**19**
(18)	*Soroca *	7	13	4	***3 ***	11	12	3	7	***4 ***	**1**
(19)	*Străşeni *	13	7	15	***11 ***	15	9	20	4	***12 ***	**12**
(20)	*Şoldăneşti *	5	16	5	***5 ***	17	1	2	18	***7 ***	**13**
(21)	*ŞtefanVodă *	10	15	21	***21 ***	21	20	17	5	***21 ***	**21**
(22)	*Teleneşti *	16	5	22	***19 ***	18	2	8	6	***5 ***	**22**

a1: land use; a2: soil quality; a3: built-up area; b1: population; b2: agriculture; b3: unemployment; b4: crime rate.

**Table 5:** Ranks of Moldova's administrative-territorial units in the decreasing order of their adaptive capacity.

ATU	Indicator's rank				Rank
	1	2	3	4	
*Anenii Noi *	8	6	17	7	***9 ***
*Bălţi *	3	22	1	14	***11 ***
*Călăraşi *	6	18	21	16	***18 ***
*Căuşeni *	18	20	10	9	***15 ***
*Chişinău *	1	9	9	1	***1 ***
*Criuleni *	17	3	13	10	***12 ***
*Donduşeni *	11	8	6	13	***10 ***
*Drochia *	16	10	7	3	***5 ***
*Dubăsari *	14	19	22	20	***22 ***
*Făleşti *	15	2	4	15	***6 ***
*Floreşti *	7	4	14	11	***7 ***
*Ialoveni *	4	15	20	8	***14 ***
*Ocniţa *	12	5	2	4	***2 ***
*Orhei *	9	2	8	18	***8 ***
*Rezina *	5	17	16	22	***17 ***
*Rîşcani *	13	7	5	2	***4 ***
*Sîngerei *	20	21	19	12	***20 ***
*Soroca *	2	13	3	5	***3 ***
*Străşeni *	10	11	18	6	***13 ***
*Şoldăneşti *	22	14	12	19	***19 ***
*ŞtefanVodă *	19	12	11	17	***16 ***
*Teleneşti *	21	16	15	21	***21 ***

1: economics; 2: agriculture; 3: medical provision; 4: housing.

**Table 6:** Ranks of Moldova's administrative-territorial units in the decreasing order of their vulnerability.

ATU	*S*	AC	Σ	Rank
*Anenii Noi *	7	13	20	***7 ***
*Bălţi *	10	12	22	***10 ***
*Călăraşi *	16	5	21	***9 ***
*Căuşeni *	18	8	26	***16 ***
*Chişinău *	5	22	27	***17 ***
*Criuleni *	6	11	17	***2 ***
*Donduşeni *	15	14	29	***19 ***
*Drochia *	3	16	19	***5 ***
*Dubăsari *	20	1	21	***8 ***
*Făleşti *	2	17	19	***6 ***
*Floreşti *	17	18	35	***22 ***
*Ialoveni *	9	9	18	***4 ***
*Ocniţa *	11	20	31	***21 ***
*Orhei *	14	15	29	***20 ***
*Rezina *	8	6	14	***1 ***
*Rîşcani *	4	19	23	***14 ***
*Sîngerei *	19	3	22	***13 ***
*Soroca *	1	21	22	***11 ***
*Străşeni *	12	10	22	***12 ***
*Şoldăneşti *	13	4	17	***3 ***
*ŞtefanVodă *	21	7	28	***18 ***
*Teleneşti *	22	2	24	***15 ***

*S*: rank of decreasing sensitivity; AC: rank of increasing adaptive capacity.

**Table 7:** The most vulnerable resources in the Dniester river basin.

Resource	Drivers of vulnerability	Adaptive capacity
Natural systems		

Human resources	The obvious impoverishment of most people, especially in rural areas, and the increasing stratification of society. A deteriorating demographic situation caused by a negative natural increase of the population and the aging of societies against the increase in morbidity and mortality. A decline in the quality of education and its incompatibility with the contemporary needs of society and, above all, the economy. Public risks associated with extreme events.	Partially retained thanks to that accumulated in previous years. For its maintenance a radical revision of Moldova's existing social and economic policies is needed.

Water resources	*The high probability* of exposure to the consequences of climate change and variability because “a river is a product of climate.” *Evident* increase of variability of the Dniester runoff regime and quantity of flow, which makes more difficult their evaluation and prognosis. Disastrous ecological conditions of small rivers, often being at risk of extinction, and reduction of their contribution to the basin's water resources. Deterioration of surface water quality due to water temperature increase, decrease in runoff, and anthropogenic pollution. *Very likely* continued decline of groundwater levels due to increased climate aridity, intense water withdrawal, and lack of the monitoring of ground water storage and quality.	Ample enough, with the anticipated maintenance or 15% increase in river flow and in the case of extending the network of water reservoirs, a competent river flow control and strict ensuring a minimum environmental runoff, as well as matching the water use with water resource availability.

Forest resources	A *likely* change in species composition and wood species' horizontal and vertical areas; the disappearance of certain hygrophilous species in the middle and lower Dniester. *A very likely *emergence of new diseases and pests. An ongoing unauthorized felling, often caused by high poverty levels.	Ample enough in the absence of human intervention in the Carpathians, but is low in Middle and Lower Dniester where intensive afforestation is needed.

Ecosystems and wetlands	*Very likely* decrease of biodiversity and the replacement of primary successions by low-productive secondary ones. *Very likely* decrease of natural habitats of indigenous species due to their drying, water quality deterioration at higher temperatures, and alien species invasion. *Likely* deficit of water supply due to the priority use of Dniester water by certain “privileged” users, for example, hydropower.	At present is low, being in essence reduced to autonomous adaptation.

Ichthyofauna	Reduction in species diversity and increase of invasive species.	Low, especially at the river mouth.

Branches of economy		

Agriculture	A *very likely* increase of aridity; more frequent and intensive droughts and extreme weather phenomena (frost, heavy rains, hail, and rainless periods), especially in the middle and lower parts of the basin. Almost complete destruction of the previous irrigation system, combined with a shortage in water resources available for irrigation. *Likely* deterioration of soil fertility due to a possible increase in soil salinity, water erosion, and landslides. *Likely* emergence and invasion of new plant pests and animal diseases. *Very likely* further depopulation of rural areas and the declining contribution of agriculture to GDP.	Low due to reduced production, rural depopulation and rural-to-urban or abroad migration, and to destruction of large farms. The absence of public subsidies that reduces the competitiveness of domestic products on local and foreign markets and export potential. Reducing the capacity and efficiency of agricultural science.

Water supply and sanitation	The *likely* falling of groundwater table and drying up of wells and springs that are main sources of water in rural area. Lack of proper diversification of water delivery to its users. *Likely* shortages of available water resources and the worsening of water quality.	Low if the present economic situation will continue.

Fish industry	*Likely* change in the fish fauna, reduction of its biodiversity, and commercial fish catches due to the disappearance or reduction of spawning grounds.	Medium in the case of the strict control of fishing and spring water releases for spawning fish.

Infrastructure	*Likely* deterioration due to both climate change direct effects (e.g., high summer temperatures or heavy rainfalls) and the lack of material resources to its maintaining.	Low due to the obvious lack of resources for maintenance and improvement.
